# Dentigerous Cysts with Diverse Radiological Presentation Highlighting Diagnostic Challenges

**DOI:** 10.3390/diagnostics12082006

**Published:** 2022-08-19

**Authors:** Alexandre Perez, Vincent Lenoir, Tommaso Lombardi

**Affiliations:** 1 Unit of Oral Surgery and Implantology, Division of Oral and Maxillofacial Surgery, Department of Surgery, University of Geneva & University Hospitals of Geneva, 1205 Geneva, Switzerland; 2 Division of Radiology, Diagnostic Department, Geneva University Hospitals, University of Geneva, 1205 Geneva, Switzerland; 3 Unit of Oral Medicine and Oral Maxillofacial Pathology, Division of Oral and Maxillofacial Surgery, Department of Surgery, University of Geneva & University Hospitals of Geneva, 1205 Geneva, Switzerland

**Keywords:** odontogenic cysts, dentigerous cyst, jaws, oral surgery, oral pathology, imaging, OPG, CBCT

## Abstract

Dentigerous cyst is an odontogenic developmental cyst arising from the pericoronal tissue of an impacted tooth, and that may exhibit various radiological aspects. The aim of this article is to present four cases of histologically confirmed mandibular dentigerous cysts to highlight diverse radiological presentations: one of classical appearance (well-limited unilocular radiolucent lesion surrounding the crown) and three which have shown radiological peculiarities (one cyst displacing the adjacent tooth, with bone but no root resorption, one cyst presenting hallmarks of infection and one multilocular cyst with thin septa). Such radiologic diversity may, on occasion, suggest a clinical aggressive lesion such as an odontogenic keratocyst or ameloblastoma. The diagnosis of dentigerous cyst requires a thorough evaluation of the clinical presentation and accurate radiological studies.

## 1. Introduction

A dentigerous cyst (DC), also known as a follicular cyst, is a cyst of non-inflammatory odontogenic origin that develops from the pericoronal tissue (dental sac or dental follicle) of an impacted tooth, either permanent or deciduous or supernumerary [1,2,3,4,5,6]. Several factors have been investigated and are known to play an important role in tooth eruption [7]. DCs represent more than 24% of the cysts of the maxilla [8,9,10]. Their incidence peaks in the third decade of life (21–30 years), followed by a gradual decrease with age. It is also slightly higher in men (sex ratio 1.5 man/L female) [2,4] and is not influenced by ethnicity [8,10].

Furthermore, 2.5 to 4% of patients with an impacted tooth develop a DC [4,8], of which 95% are associated with a permanent tooth and 5% with a supernumerary tooth [2,11,12]. Regarding localization, 74% of DCs localize in the mandible and 26% in the maxilla with a symmetrical left/right distribution [13,14,15,16].

Bilateral lesions are extremely rare, and when occurring, association with development anomalies and syndromes, such as mucopolysaccharidosis or cleidocranial dysplasia, should be suspected [1,2,5].

Clinically, DCs are often asymptomatic but may occasionally cause swelling and dental displacement [8,9,14,15]. More rarely, a DC may be accompanied by pain caused by superposed infection or paresthesia when mechanical compression on a nerve occurs [11]. DCs are most often diagnosed incidentally during an oral check-up, and the panoramic dental X-ray (OPG) is generally the most frequent diagnostic imaging technique carried out.

Radiologically, DCs usually appear as well-defined unilocular radiotransparent homogeneous lesions with a round or ovoid shape attached to the cementoenamel junction of an impacted tooth, usually third molars, and largely in the mandible [9,11,12].

The borders of the lesion may appear sclerotic and are less well-defined when the cyst becomes infected. When the cyst is large, it may displace or even resorb adjacent dental roots or may induce bone remodelling. However, the bone cortex usually remains intact.

In this article, we present four cases of DC to highlight the diverse radiological presentation: one of classical appearance (case #2) and three which have shown radiological peculiarities (case #1, cyst displacing the adjacent tooth, with bone but no root resorption; case #3, cyst presenting hallmarks of infection; case #4, multilocular cyst with thin septa).

## 2. Case Series

All the cases presented refer to lower third molars.

Case #1:

A 30-year-old male in good general health consulted the Oral Surgery and Implantology Unit of the Geneva University Hospitals for pain experienced for two days localized in the left lower quadrant of the mandible, with no fever nor ear, nose, and throat symptoms.

On extraoral examination, palpation at the angle and the horizontal branch of the mandible was painful, and so was intraoral palpation at the vestibular level from tooth #37 to the left ascending branch of the mandible.

On dental examination, tooth #37 showed sensibility positive test, slightly painful percussion, and a pocket depth on probing of more than 9 mm localized distally and associated with suppuration.

**Figure 1:**
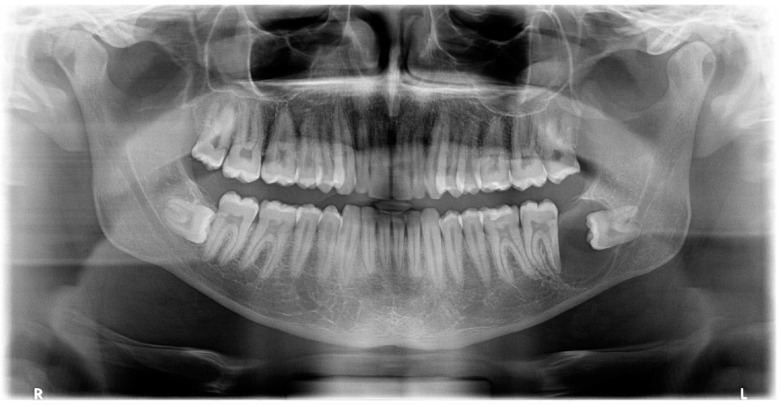
Panoramic X-ray of case #1.

Panoramic radiography revealed the presence of a pericoronal radiolucent lesion around the crown of an impacted tooth #38 (horizontally positioned with mesial orientation) (Figure 1). The lesion measuring 27 × 22 mm was well defined and unilocular, surrounded by a thin sclerotic bone. It overlapped with the distal root of tooth #37, causing adjacent bone resorption but with no signs of root resorption. The cyst seemed to interfere with the inferior alveolar canal (IAC), which appeared displaced caudally by the lesion. A presumptive diagnosis of CD was made.

**Figure 2:**
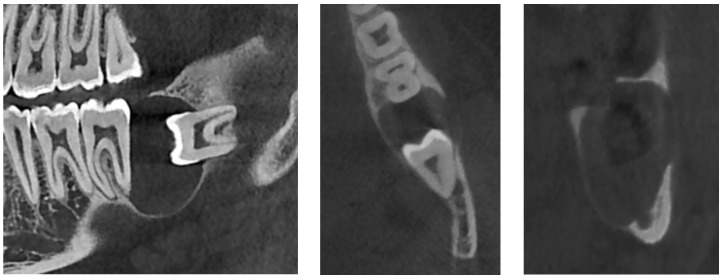
CBCT X-ray of case #1.

An additional cone-beam computed tomography (CBCT) X-ray examination confirmed the presence of the large pericoronal cystic lesion attached to the cementoenamel junction of tooth #38 (Figure 2). We observed a bone expansion accompanied by slight thinning of the mandibular cortex on the vestibular and lingual sides. The cyst was in contact with the upper wall of the IAC, which was thinned and caudally displaced, but of normal width. This lesion had an approximately 8 mm opening on the mucosal alveolar crest and extended to the distal root of tooth #37 without signs of associated root resorption. 

**Figure 3:**
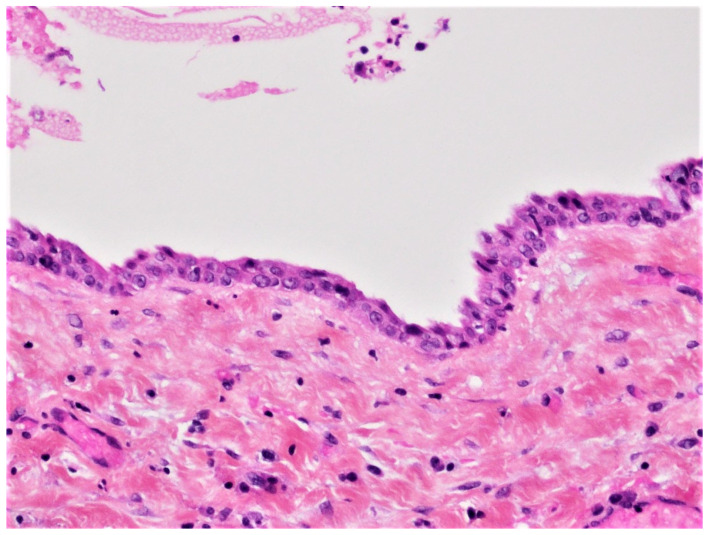
Histopathological examination of the enucleated specimen from case #1 showing an uninflamed fibrous cyst wall lined by a thin cuboidal epithelial lining (H&E, ×40).

**Figure 4:**
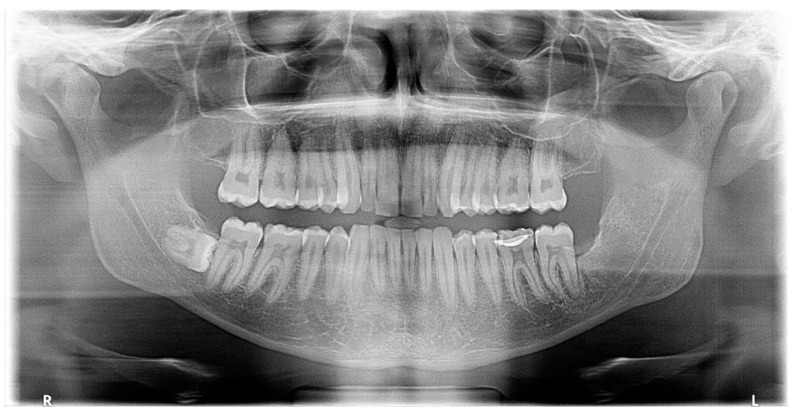
Panoramic X-ray of case #1 at one-year follow-up.

There was also no periosteal reaction, pathological fracture, evident signs of sequestration or sclerosis of the surrounding bone, nor infiltration of the perimandibular soft tissues. Tooth #38 had two roots, and the mesial root came into contact with the lingual wall of the IAC. The radiological appearance evoked a DC. Differential diagnoses included odontogenic keratocyst and unicystic ameloblastoma. The lesion was enucleated in toto together with tooth #38 under local anesthesia. The diagnosis of DC was confirmed by histopathological examination (Figure 3). The patient was followed for four years. At 12 months follow-up, OPG showed satisfactory healing of the enucleation cavity and tooth #37 was still vital (Figure 4). The patient was followed-up for 4 years with no signs of recurrence.

Case #2:

A 38-year-old female in good general health was referred by her orthodontist for diagnosis and treatment of a lesion in the right lower quadrant of the mandible discovered incidentally on an OPG X-ray performed prior to orthodontic treatment. The oral examination was unremarkable, and the patient was asymptomatic.

**Figure 5:**
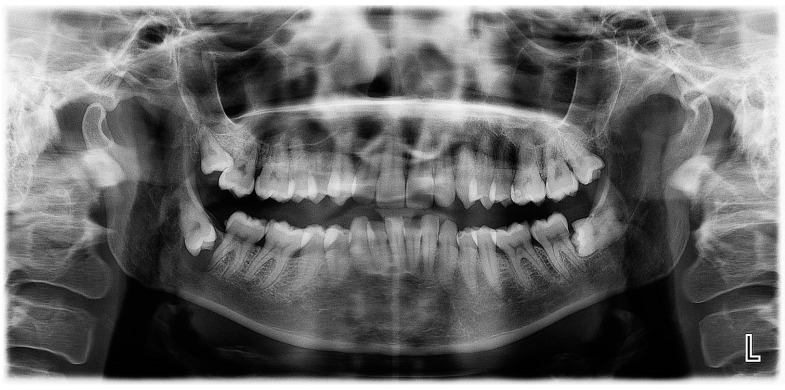
Panoramic X-ray of case #2.

A new OPG X-ray revealed the presence of a pericoronal radiolucent lesion on an impacted tooth #48, which was inverted in the mesio-caudal direction (Figure 5). The lesion was well defined, measured 13 × 15 mm, and was surrounded by a thin sclerotic border overlapping with the distal root of tooth #47, which appeared slightly resorbed. The cyst also overlapped with the IAC, whose walls could not have been well-identified during this exam. The presumptive diagnosis was a DC.

**Figure 6:**
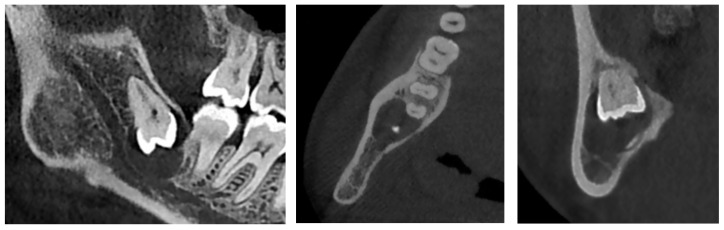
CBCT X-ray of case #2.

On the complementary CBCT X-ray, it was observed an impacted tooth #48 in horizontal/inverted orientation in the mesio-caudal direction (Figure 6). The two roots were not in direct contact with the right IAC, and the tooth crown did not come into direct contact with the root of tooth #47. The pericoronal space was markedly enlarged, 13 mm wide, 17 mm high, and 15 mm long, compatible with a DC. The visible resorption of the distal root of tooth 47 made a differential diagnosis of odontogenic keratocyst less likely. The lesion was associated with an alveolar crest dehiscence of over 4 mm in length and thinned lingual cortex. The lesion displaced the IAC caudally, causing loss of visibility of its wall.

**Figure 7:**
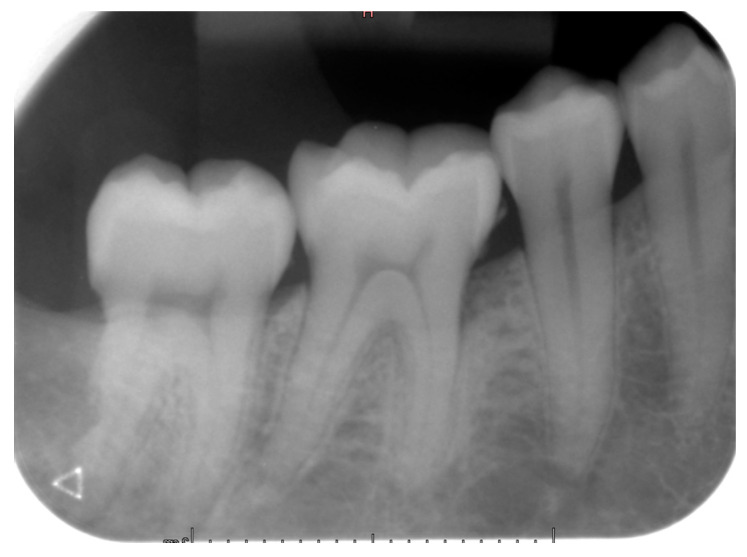
Intraoral X-ray of case #2 at one-year follow-up exam.

Teeth #18, 28, 38 and 48 were extracted, and the pericoronal lesion in tooth #38 was enucleated in toto under local anesthesia. The diagnosis of DC was confirmed by histopathological examination. The patient was followed-up for two years. At 12 months of control, an intraoral X-ray showed good healing of the enucleation cavity of the follicular cyst (Figure 7). Clinically, tooth #47 remained vital and asymptomatic.

Case #3:

A 46-year-old female in good general health consulted the Oral Surgery and Implantology Unit of the Geneva University Hospitals for the management of a lesion in the left lower quadrant of the mandible discovered fortuitously on an OPG X-ray performed by her dentist. The patient had a history of episodic pain in this area over several months, which she treated intermittently with self-medicated painkillers and anti-inflammatory drugs.

**Figure 8:**
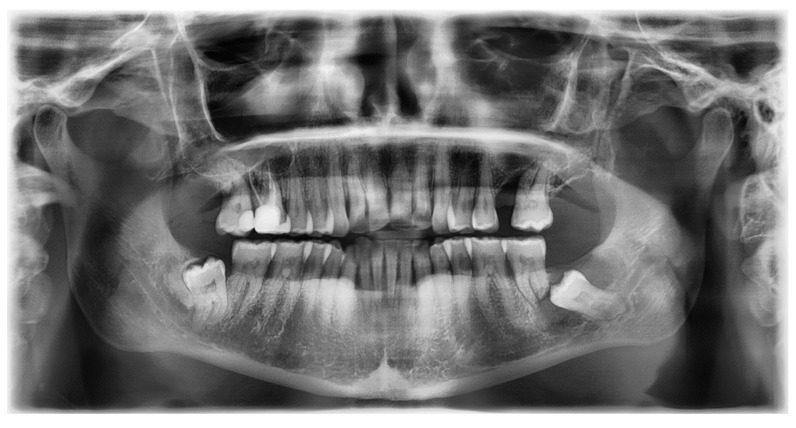
Panoramic X-ray of case #3.

The oral clinical examination was unremarkable, and the patient was asymptomatic. On the OPG, a pericoronary radio-transparent lesion around impacted tooth #38 was discovered (Figure 8). The involved tooth was oriented mesially. The lesion was poorly delimited, measured 24 × 10 mm, and stretched to the apparently resorbed distal root apex of tooth #37. The roots of tooth #38 clearly overlapped with the IAC, raising suspicion of interference. The OPG images were indicative of a DC with possible secondary infection and perilesional sclerosing osteitis. 

**Figure 9:**
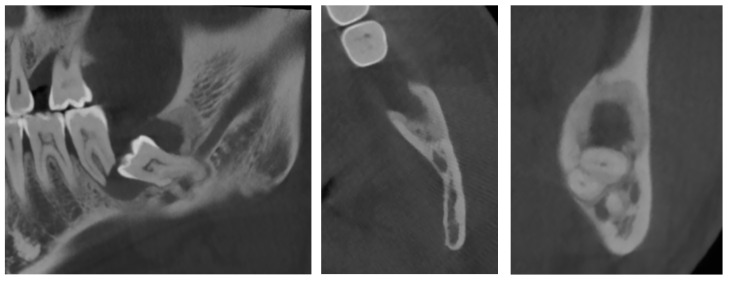
CBCT X-ray of the case #3 patient.

The complementary CBCT examination found that tooth #38 was directed mesially and slightly lingually (Figure 9) and that it had three roots, with the deformed IAC tortuously passaging between them. The crown of tooth #38 was close to the distal root of tooth #37, whose apex was strongly resorbed. The pericoronal osteolytic lesion measuring 24 × 10 × 10 mm extended from the crown-root junction of tooth #38 to the partially resorbed distal root of tooth #37. There was bone dehiscence of 12 mm on the alveolar crest and of 5 mm on the vestibular cortex. The borders of the lesion were in places irregular and strongly sclerotic, indicating a superimposed infection. There were no signs of bone sequestration, periosteal reaction or associated fracture. These findings supported the initial diagnosis of DC.

Teeth #37, 38 and 48 were extracted under local anesthesia. The lesion on tooth #38 was submitted for histopathological examination, which confirmed the diagnosis of DC.

Case #4:

A 59-year-old male was referred to the Oral Surgery and Implantology Unit of the Geneva University Hospitals for a comprehensive oral examination and dental care prior to the start of radio- and chemotherapy for squamous cell carcinoma of the floor of the mouth. The patient was taking Rivotril^®^ for epilepsy, Beloc ZOC^®^ and Atacand^®^ for high blood pressure and Crestor^®^ for hyperlipidemia. He was a heavy smoker (70 pack year) and consumed 60 g of alcohol a day.

The extraoral clinical examination was unremarkable. Intraorally, the mucosa of the hard and soft palate as well as the lingual mucosa showed keratotic dots; on the floor of the mouth, there was a 3 cm long and 1 cm wide ulceration; the oral vestibule presented a smoker keratosis. Oral hygiene was poor with plaque and tartar deposits; the patient had severe periodontal disease with grade III furcation involvement of teeth #46, 26 and 16, polycaries of teeth #16, 14, 13, 12, 23, 25, 46, 45, 43, 42, 41, 32, 33, 34 and 35; teeth #15, 14, 11, 22, 27, 47, 36 and 37 were absent.

**Figure 10:**
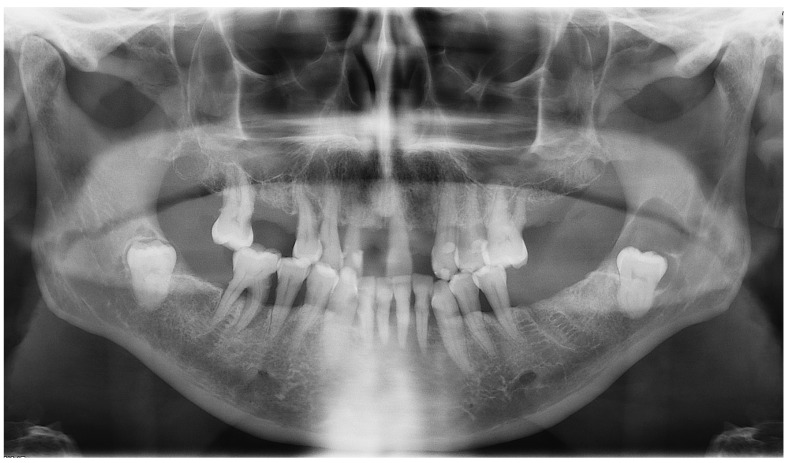
OPG X-ray of case #4.

On an OPG X-ray, teeth #38 and 48 were impacted and distally orientated (Figure 10). Tooth #38 showed a radiotransparent lesion measuring 20 × 15 mm, with a well-defined border, multilocular appearance, and a thin septum, most compatible with a DC versus ameloblastoma versus odontogenic keratocyst.

**Figure 11:**
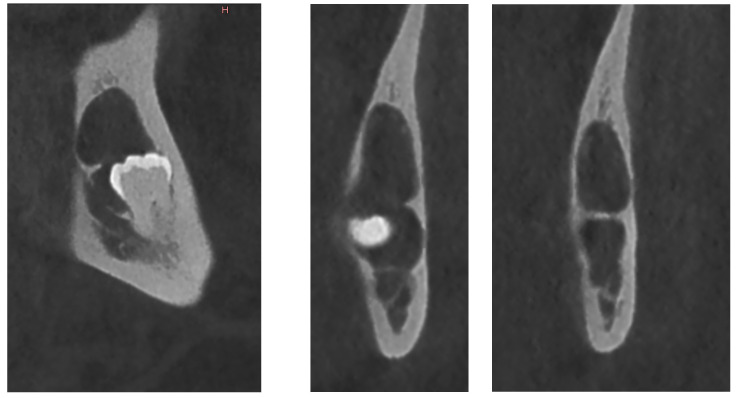

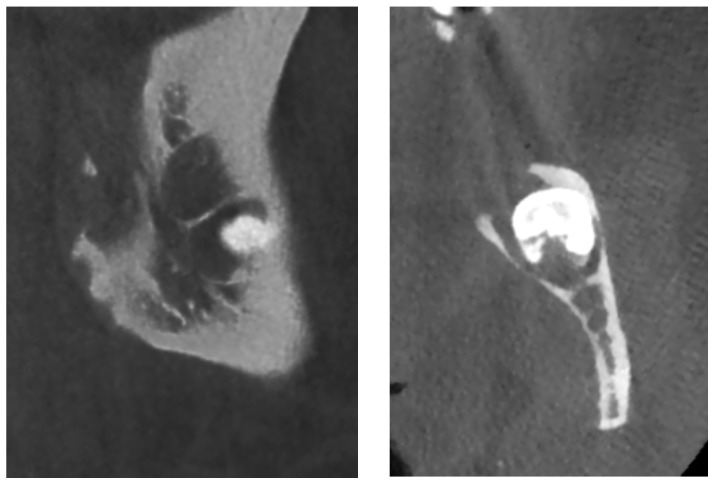
CBCT X-ray of case #4.

On CBCT X-ray, the distal root of the impacted tooth #38 was in contact with the upper wall of the left IAC, generating a discreet deformation of the canal (Figure 11). The cyst showing a multilocular aspect was located adjacent to the crown of tooth #38 and seemed to be attached to the tooth neck. The lesion extending slightly to the ascending branch of the mandible measured 17 mm in height, 21 mm in length and 10 mm in width. In its caudal part, the lesion was in contact with the upper wall of the IAC over about 13 mm. The wall was thinned but with little impact on its internal diameter. The vestibular and lingual cortex were also strongly thinned focally. There was no periosteal reaction or fracture at the mandibular angle. On the bases of these radiological findings, the differential diagnosis included DC, ameloblastoma, and odontogenic keratocyst. 

**Figure 12:**
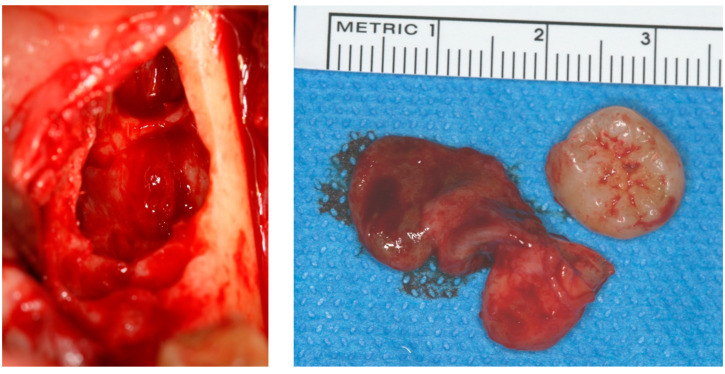
Avulsion of tooth 38 and in toto enucleation of the cyst in case #4.

The lesion was enucleated in toto, and the teeth extracted (Figure 12) under general anesthesia, during which the ENT and maxillofacial surgeons proceeded with the placement of a mandibular osteosynthesis plate, followed by non-interruptive mandibulectomy, and finally, pelvi-glossectomy with tracheostomy and reconstruction with an anterolateral thigh flap and neck dissection. Histopathological examination of the cystic lesion allowed to diagnose a DC. The patient was followed regularly for two years, and no recurrence was observed. 

**Figure 13:**
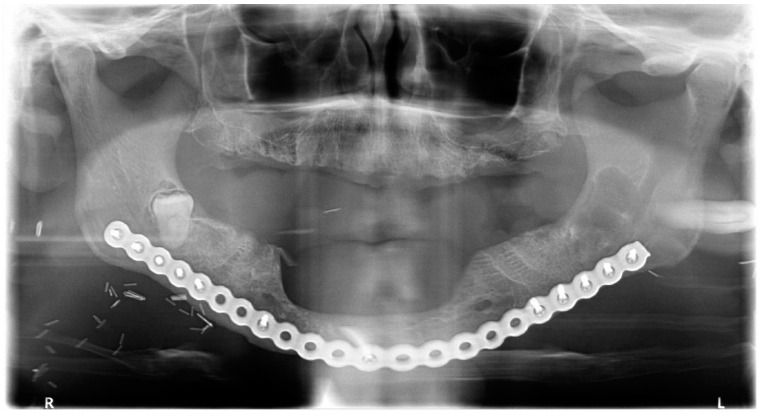
CBCT X-ray of case 4 at one year follow-up.

Healing was uneventful and the patient had no complaints. Radiologically, at the 6- and 12-month follow-up (Figure 13), there was no reossification at the cystectomy and tooth 38 sites, which could be explained by a side effect of postoperative radiotherapy and chemotherapy. However, there were no signs of enlargement of the residual bone cavity, suggesting a recurrence of DC. 

## 3. Discussion

**Figure 14:**
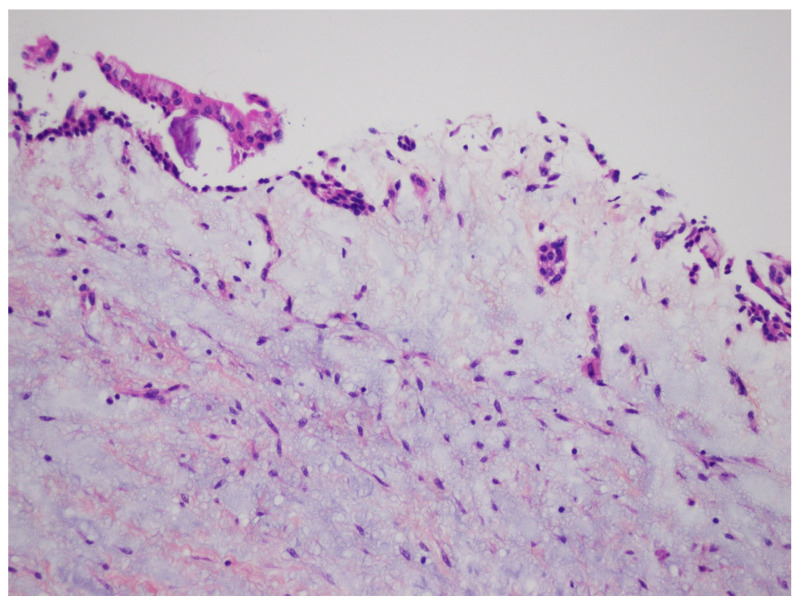
Dental follicle partly lined by reduced enamel epithelium (H&E, ×20).

DC is the second most common odontogenic cyst occurring in association with an unerupted tooth. It develops from the accumulation of fluid between the reduced enamel epithelium of the dental follicle and the crown of an unerupted tooth (Figure 14). The microscopic features of DC are dependent whether it is not inflamed or inflamed [8,9,17,18]. In the case of non-inflamed DC, the epithelial lining is formed by two to four layers of cuboidal/squamous non-keratinizing cells without rete ridges and a flat epithelium-connective tissue interface (Figure 3). The cyst wall consists of fibrous or fibro-myxoid tissue containing considerable glycosaminoglycan ground substance. Small islands or cords of inactive-appearing odontogenic epithelial rests are usually present within the connective tissue. 

**Figure 15:**
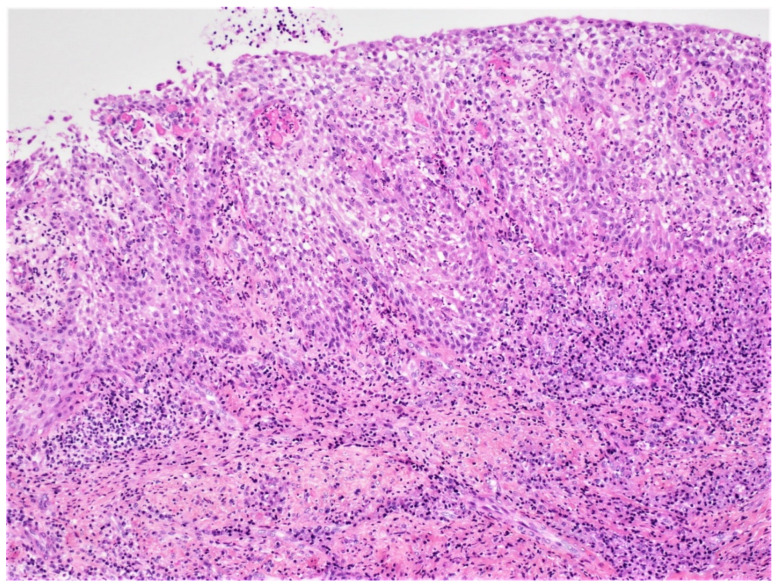
Non-keratinized spongiotic squamous epithelium presenting hyperplastic rete ridges. The fibrous wall contains a lymphoplasmocytic infiltrate (H&E, ×10).

In the case of inflamed DC, histopathologic examination reveals a fibrous-connective tissue wall with a variable infiltration of chronic inflammatory cells and, on occasion, cholesterol clefts. The cyst is lined in part or entirely by non-keratinizing squamous epithelium, which shows varying amounts of hyperplasia with the development of elongated and interconnected rete ridges. These features may lead to a misdiagnosis of radicular cyst (Figure 15). Mucus cells or, rarely, ciliated columnar cells may be observed in the epithelial lining.

Although the definitive diagnosis of cystic lesions requires histopathological analysis, clinical and radiological examinations are of paramount importance in establishing the differential diagnosis [8,9]. In the case of DC, the presumptive preoperative diagnosis is usually based on an analysis of OPG images [1,9,10]. Certain radiological signs are specific, while others are non-specific or variable. The radiological peculiarity of DCs, which facilitates their recognition, is the fact that the lesions are attached to the cementoenamel junction and surround the tooth crown of an impacted tooth [8,9,10]. The center of the cyst may be located above or below the crown, except when the tooth is not in an upright position or when the cyst orients laterally during its development. Other radiological signs, less specific to DC, are the boundaries of the lesion: generally, well-defined, rounded, a thin sclerotic margin [4,8,11], and the radiolucent homogeneous, unilocular internal appearance of the lesion [4,5,6,7,8,9]. On the other hand, the effects on the surrounding anatomical structures (adjacent teeth, bone cortex, IAC, nasal cavity, and maxillary sinus floor) can be variable and include discharge, displacement, and expansion or resorption [8,9,12]. CBCT exam is very important in the diagnosis of DC. Their appearance on CBCT is similar to panoramic radiography, however, this exam provides more precise information on the size, position, and relationship of the lesion to the surrounding structures. In classical helical computed tomography, the content of a DC typically appears as low density on CBCT (liquid-like). The 3-dimensional analysis of the lesion enabled by CBCT provides precise and important information for clinicians developing a treatment strategy and surgical approach. A superior projection of the lesion on the nasal sinus cavities and displacement of the IAC can be accurately highlighted. Similarly, the expansion of the vestibular or lingual cortex is easily evaluated. Magnetic resonance imaging (MRI) has a very limited role in the diagnostics of this pathology, helping to distinguish these lesions from other cystic bone lesions only when the presentation is atypical. The contents of DC typically appear liquid-like on MRI (in hypointensity T1 and hyperintensity T2) and lack solids’ high-contrast partitions, except for occasional fine peripheral contrast enhancement when the cyst is infected.

In the cases presented here, the presumptive radiological diagnosis of DC was confirmed by histopathological examination. However, in the OPG and CBCT images, the lesions varied in size, contour appearance, relationship, and effects on the surrounding anatomical structures. Differential radiological diagnoses differed from case to case and are summarized in Table 1. 

Radiologically differential diagnosis of DC is mainly made with hyperplastic dental follicle, odontogenic keratocyst, and unicystic ameloblastoma. The following characteristics point toward a DC: neighboring tooth infiltration, bubble-like cortex, and the pericoronal space thicker than 5 mm [1,4,8,9,16]. An odontogenic keratocyst in pericoronal localization (about 21% of cases) can be considered in the differential diagnosis, especially in large lesions, but it causes bubble-like cortex and root resorption less frequently, has a slightly denser content in radiographic images [19,20,21,22,23], and never really attaches to the cementoenamel junction [8]. A unicystic ameloblastoma or an ameloblastic fibroma cannot be differentiated radiologically due to the absence of an internal structure [8,21]. A radicular cyst located at the apex of a deciduous tooth and surrounding the crown of the underlying permanent tooth can also resemble a cyst [8,24]. An odontogenic adenomatoid tumor or a calcified odontogenic cyst can also resemble a DC [8,24].

In this case series, the most likely differential diagnoses were keratocyst and unicystic ameloblastoma. The radiological findings that influenced the differential diagnosis were mainly the effects of the lesion on the adjacent anatomical structures (root resorption vs. infiltration) and the multilocular contour (case #4). On the other hand, although the 3D CBCT X-ray allowed us to appreciate the lesion volume and the relationship between the lesion and the surrounding structures, the differential diagnosis remained the same as the one deduced based on the OPT.

For cases #2 and 3, the presumptive diagnosis of DC was quite high because of the classical appearance and the resorption of the adjacent roots made it possible to exclude keratocyst. This was in contrast to case #1, where the teeth/roots were displaced. Case #4, particularity, was the presence of a well-defined multilocular contour and the presence of septa, both of which, however, did not exclude the possibility of a keratocyst and ameloblastoma.

When the DC is of large size, it can predispose the patient to pathological infections or fractures [8,9]. Rare cases of ameloblastic transformation in DC have been described in the literature [21]. 

The treatment of DC is represented by enucleation followed by curettage. It has been proposed that natural polymers may favorably help bone regeneration [25]. 

The use of local antibiotics after the enucleation of the cyst has been advocated [26], as well as the use of analgesics [27]. 

## 4. Conclusions

We have presented four examples of radiological presentation of DCs. Diagnosing DC lesions involved multiple aspects. Although it is most often based on OPT images where it appears as a radiolucent lesion surrounding an impacted tooth crown, other characteristics of the lesion should be taken into account since they may lead to a different differential diagnosis. 

Limitations of this study is the observational nature based on a small number of cases.

## Figures and Tables

**Table 1:** Summary of the features of the presented cases.

Case No	Impacted Tooth	DC Size	Contour	Relationship with Dental Anatomical Structures	Relationship with Anatomical Bone Structures	Differential Diagnosis
1	38, horizontal position with mesial orientation	27 × 22 mm (OPG and CBCT)	Well defined, thin sclerotcs border of	Adjacent teeth: - Tooth 37, distal bone resorption - No root resorption - Discharge effect (distalized dental axis) - Tooth vital	IAC: - Signs of interference - Strongly thinned wall - Caudally displaced, no narrowing of the canal Cortex: - Bubble-like vestibular and lingual cortex (infra-millimeter thinning)	- Dentigerous cyst - Keratocyst - Unicystic ameloblastoma
2	48, inverted position with mesio-caudal orientation	13 × 15 mm (OPG) 13 × 17 × 15 mm (CBCT)	Well defined, thin sclerotic border	Adjacent teeth: - Severely resorbed apex of the distal root of tooth 47 - Tooth vital	IAC: - Signs of interference - Caudally displaced - Cranial cortex discontinuity Cortex: - Bubble-like lingual cortex - 4 mm alveolar crest dehiscence	- Dentigerous cyst (root resorption means keratocyst was less likely)
3	38, mesial	24 × 10 mm (OPT) 24 × 10 × 10 mm (CBCT)	In places irregular, strongly sclerotic (sign of superinfection)	Adjacent teeth: - Suspicion of resorption of root apex 37 (OPT) - Strongly resorbed root apex 37 (CBCT) - Tooth 37 necrotic	IAC: - Apeces 38 interfering with IAC - Internal canal deformation Cortex: - 12-mm alveolar crest dehiscence lingually and 5-mm vestibulary	- Infected dentigerous cyst (root resorption means keratocyst was less likely)
4	38, distal	20 × 15 mm (OPG) 17 × 21 × 10 mm (CBCT)	Well defined, multilocular, thin septa, absence of periosteal reaction	-	IAC: - Contact of superior edge (13 mm) - IAC thinned wall - No canal deformation Cortex: - Bubble-like lingual and vestibular cortex	- Dentigerous cyst - Ameloblastoma - Keratocyst

## Data Availability

Not applicable.
